# Supplementary material for: Comprehensive Transcriptomic and Metabolic Profiling of Agrobacterium-tumefaciens-Infected Immature Wheat Embryos
Source: Int J Mol Sci. 2023 May 8;24(9):8449. doi: 10.3390/ijms24098449 (PMC10179373; doi:10.3390/ijms24098449)
Supplement: Supplementary file 1 [file ijms-24-08449-s001.zip › Supplementary file S1.pdf]

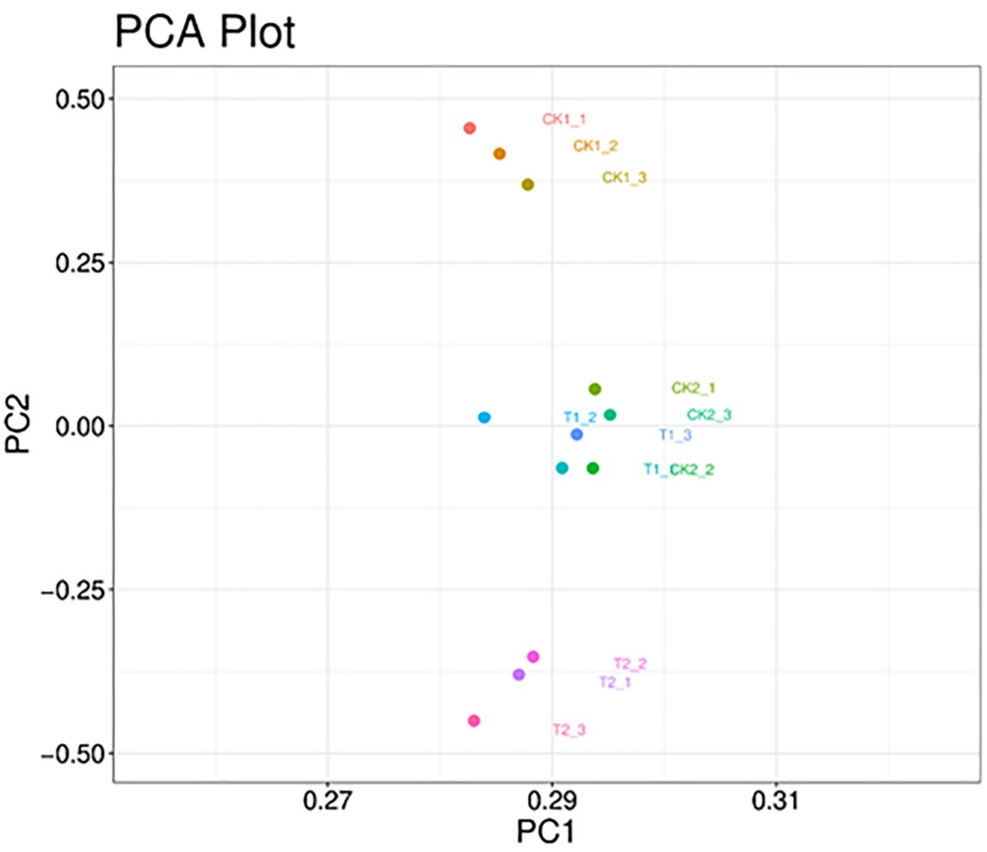

Figure S1. The PCA analysis of RNA-seq data

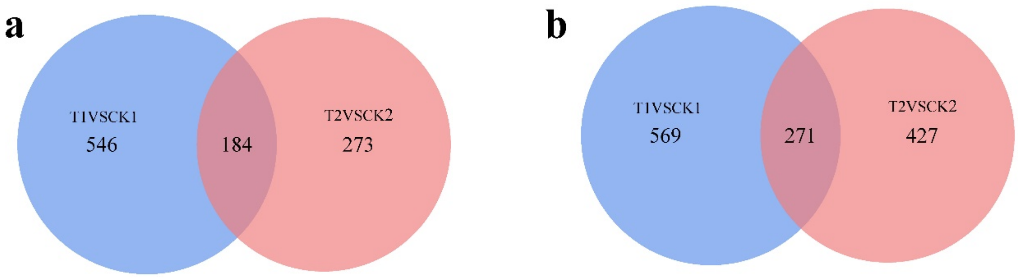

Figure S2. (a) Venn diagram of upregulated DEGs revealed in pair-wise comparisons; (b)Venn diagram of downregulated DEGs revealed in pair-wise comparisons

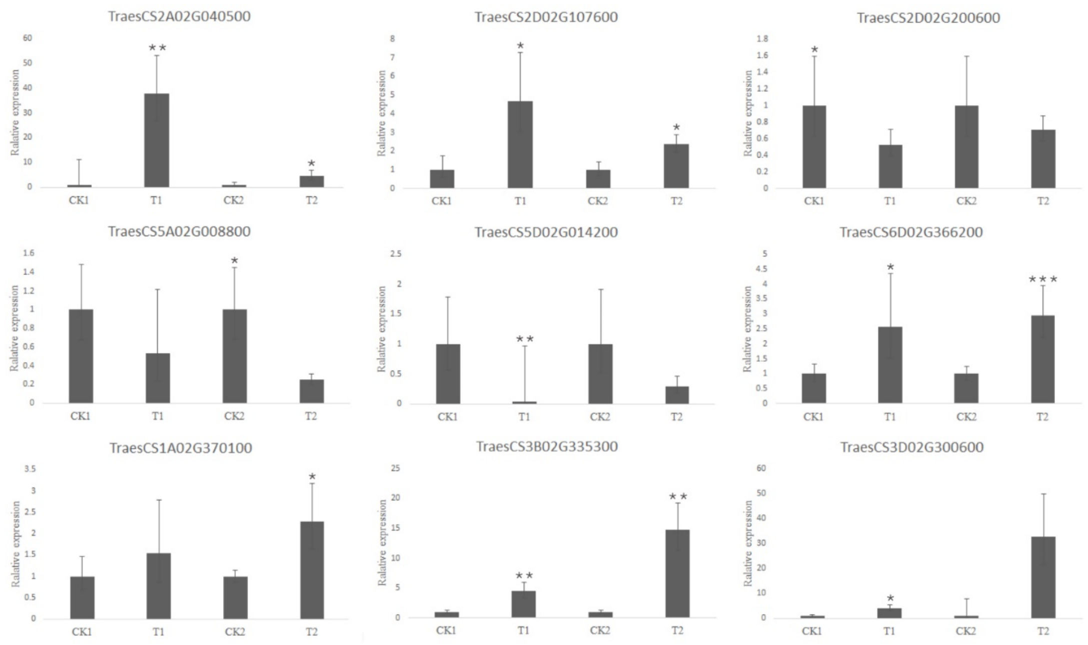

Figure S3. qRT-PCR validation of the DEGs of young embryos infected with *Agrobacterium* tumefaciens. * indicates significant difference at $p < 0.05$, ** indicates significant difference at $p < 0.01$, and *** indicates significant difference at $p < 0.001$.

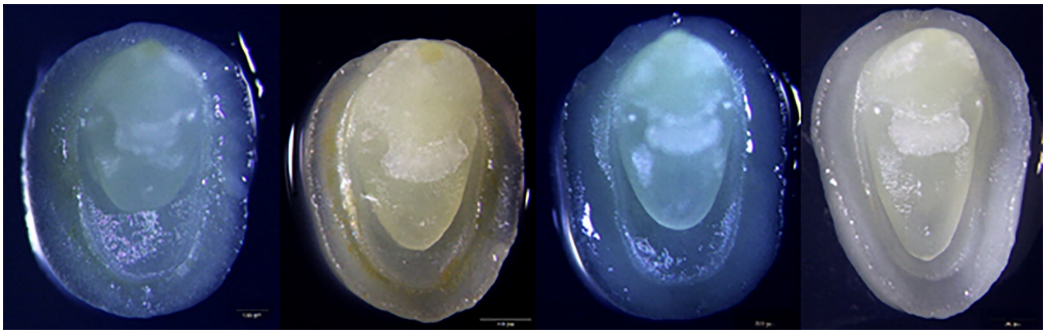

Figure S4. Wheat immature embryo of 2-2.5 mm in length used in the study
