# Supplementary material for: Comprehensive Transcriptomic and Metabolic Profiling of Agrobacterium-tumefaciens-Infected Immature Wheat Embryos
Source: Int J Mol Sci. 2023 May 8;24(9):8449. doi: 10.3390/ijms24098449 (PMC10179373; doi:10.3390/ijms24098449)
Supplement: Supplementary file 1 [file ijms-24-08449-s001.zip › Supplementary file S3.pdf]

The following methods are derived from PureWheat technique developed by Ishida et al. (Ishida, Y.; Tsunashima, M.; Hiei, Y.Komari, T. Wheat (Triticum aestivum L.) transformation using immature embryos. *Methods in molecular biology (Clifton, N.J.)* **2015**, *1223*, 189-198.)

## Preparation of Inoculum

1. Culture A. tumefac iens in 10 ml MG/L medium overnight at 28 °C with vigorous shaking.
2. Collect bacteria by centrifugation and resuspend at the cell density of 0.4 of A 660 in WLS-inf medium.

3.1.2 Preparation of Immature Embryos

1. Collect immature seeds at the right developmental stage from panicles about 14 days after anthesis (DAA)
2. Remove glume, lemma, and palea with forceps.
3. Sterilize immature seeds with 70 % ethanol for 1 min and 1 % sodium hypochlorite for 10 min, and then wash 3 times with sterilized distilled water.
4. Isolate immature embryos from the immature seeds on the clean bench.
5. Transfer the embryos into 2.0 ml of WLS-liq medium in a 2.0 ml micro-centrifuge tube at room temperature, each tube contains 25 embryos.
6. Invert the micro-centrifuge tube several times and remove the medium.
7. Add 2.0 ml of WLS-liq medium.

Agrobacterium multiplication (MG/L): Add 5 g mannitol, 1 g L-glutamic acid, 250 mg $KH_2PO_4$, 100 mg NaCl, 100 mg $MgSO_4 \cdot 7H_2O$, 5 g tryptone, 2.5 g yeast extract, and 1 μg biotin to 700 ml of distilled water and make up the volume to 1,000 ml. Adjust pH to 7.0. Sterilize using a 0.22 μm cellulose acetate fi lter and store at 4 °C.

## Pretreatment and Cocultivation

1. Centrifuge the micro-centrifuge tube with a fi xed-angle rotor with a maximum radius of 83 mm at 20,000 × g at 4 °C for 10 min
2. Remove the medium from the micro-centrifuge tube and add 1.0 ml inoculum.
3. Invert the micro-centrifuge tube frequently for 30 s.
4. Incubate at room temperature for 5 min.
5. Transfer the immature embryos to WLS-AS medium with the scutellum side up
6. Immature embryos were collected and named T1 or CK1 after one day incubation

in 23°C darkness, Immature embryos were collected and named T2 or CK2 after incubation in the dark for two days.

## Stock Solutions

1. LS major salts (10×): Dissolve 19.0 g $KNO_3$, 16.5 g $NH_4NO_3$, 4.4 g $CaCl_2 \cdot 2H_2O$, 3.7 g $MgSO_4 \cdot 7H_2O$, and 1.7 g $KH_2PO_4$ in 900 ml distilled water and fill to 1,000 ml. Store at 4 °C.
2. Ethylenediamine-tetraacetic acid-iron (FeEDTA, 100×): Dissolve 2.78 g $FeSO_4 \cdot 7H_2O$ in 900 ml of hot distilled water and add 3.73 g ethylenediamine- N, N, N ′, N ′-tetraacetic acid, disodium salt. Cool and fill to 1,000 ml. Store at 4 °C.
3. LS minor salts (100×): Dissolve 2.23 g $MnSO_4 \cdot 5H_2O$, 1.06 g $ZnSO_4 \cdot 7H_2O$, 620 mg $H_3BO_3$, 83 mg KI, 25.0 mg $Na_2MoO_4 \cdot 2H_2O$, 2.5 mg $CuSO_4 \cdot 5H_2O$, and 2.5 mg $CoCl_2 \cdot 6H_2O$ in 900 ml of distilled water and fill to 1,000 ml. Store at 4 °C.
4. MS vitamins (100×,): Dissolve 10 g myoinositol, 0.2 g glycine, 100 mg thiamine hydrochloride, 50 mg pyridoxine hydrochloride, and 50 mg nicotinic acid.
5. Modified LS vitamins (100×): Dissolve 10 g myoinositol, 100 mg thiamine hydrochloride, 50 mg pyridoxine hydrochloride, and 50 mg nicotinic acid in 900 ml of distilled water and fill to 1,000 ml. Store at 4 °C.

## Media Composition

1. Agrobacterium multiplication (MG/L): Add 5 g mannitol, 1 g L -glutamic acid, 250 mg $KH_2PO_4$, 100 mg NaCl, 100 mg $MgSO_4 \cdot 7H_2O$, 5 g tryptone, 2.5 g yeast extract, and 1 μg biotin to 700 ml of distilled water and make up the volume to 1,000 ml. Adjust pH to 7.0. Sterilize using a 0.22 μm cellulose acetate filter and store at 4 °C.
2. Embryo collection (WLS-liq): Add 10 ml of the 10× LS major salts, 1 ml of 100× FeEDTA, 1 ml of 100× LS minor salts and 1 ml of 100× MS vitamins, 10 g glucose, and 0.5 g 2-(N-morpholino) ethanesulfonic acid (MES) to 700 ml of distilled water and make up the volume to 1,000 ml. Adjust pH to 5.8. Sterilize using a 0.22 μm cellulose acetate fi lter and store at 4 °C.
3. Inoculum (WLS-inf): WLS-liq plus 100 μM acetosyringone.
4. Cocultivation (WLS-AS): WLS-inf plus 0.85 mg/l $AgNO_3$,1.25 mg/l $CuSO_4 \cdot 5H_2O$, and 8 g/l agarose.
